# Chromosomal diversity in three species of *Lycosa* Latreille, 1804 (Araneae, Lycosidae): Inferences on diversification of diploid number and sexual chromosome systems in Lycosinae

**DOI:** 10.1590/1678-4685-GMB-2020-0440

**Published:** 2022-01-24

**Authors:** Analiza Fernanda Cavenagh, Matheus Pires Rincão, Felipe Cordeiro Dias, Antonio Domingos Brescovit, Ana Lúcia Dias

**Affiliations:** 1 Universidade Estadual de Londrina, Departamento de Biologia Geral, Laboratório de Citogenética Animal (LACA), Londrina, PR, Brazil.; 2 Instituto Butantan, Laboratório de Coleções Zoológicas, São Paulo, SP, Brazil.

**Keywords:** FISH, heterochromatin, rDNA, sex chromosomes, spiders

## Abstract

*Lycosa* is one of the most speciose genera in Lycosidae, including species with different sexual chromosome systems (SCS). We carried out cytogenetic analyses in three species of *Lycosa*, revealing that *L. erythrognatha* and *L. sericovittata* share 2n ♂ = 22 and SCS X_1_X_2_0 while *L.* gr*. nordenskjoldi* presents 2n ♂ = 19 and SCS XO, composed only of acrocentric chromosomes. All species shared pericentromeric heterochromatin. Nonetheless, one specimen of *L. sericovittata* carried two chromosomes with terminal heterochromatin and *L*. gr. *nordenskjoldi* showed four chromosomes with interstitial heterochromatin plus another chromosome with terminal C-bands. The pericentromeric heterochromatin of all species as well as the terminal heterochromatic blocks in *L. sericovittata* were CMA_3_^+^ *.* The 18S rDNA sites varied in number and type of bearing chromosomes both at inter and intrapopulational levels, with the highest variation in *L.* gr*. nordenskjoldi*. These differences may be related to gene dispersal due to the influence of transposition elements and translocation events. Despite these variations, all species shared ribosomal sites in pair 5. This study demonstrated intra and interspecific chromosomal variability of *Lycosa*, suggesting that chromosomal rearrangements are related to the diversification of diploid number and SCS in this group of spiders.

## Introduction

Members of the Lycosoidea superfamily belong to the Entelegynae clade in Araneomorphae, composing a highly diverse group, with more than 6,000 species and 422 genera (World Spider Catalog, 2021) distributed in seven families: Ctenidae, Lycosidae, Oxyopidae, Pisauridae, Psechridae, Thomisidae, and Trechaleidae (Wheeler et al., 2017). Lycosidae comprises 2,400 species and 125 genera, representing nearly 50% of the species described in Lycosoidea (World Spider Catalog, 2021). 

Although Lycosidae is one of the most studied families cytogenetically, chromosomal data are only available in 5% of the described species, which indicates a large gap in knowledge about carioevolutionary trends in this large and widespread group of spiders (Araujo *et al*, 2021). So far, the diploid number reported in species of this family ranges from 18 to 30 chromosomes, mostly acrocentric/telocentric, with a predominance of 2n ♂ = 28 (reported in 62 of the 120 cytogenetically analyzed species). The sex chromosome system (SCS) X_1_X_2_0/X_1_X_1_X_2_X_2_ is present in 94% of the described karyotypes in Lycosidae (Araujo *et al*., 2021), and variations are restricted to the occurrence of X0 systems as observed in *Lycosa barnesi* Gravely, 1924, *Wadicosa quadrifera* (Gravely, 1924) (Srivastava and Shukla,1986), *Lycosa* gr. *nordenskjoldi* Tullgren, 1905, *Hogna sternalis* (Bertkau, 1880) (Araujo *et al*., 2015); X_1_X_2_X_3_0 in *Lycosa* sp. (group *thorelli*) (Postiglioni and Brum-Zorrilla, 1981), and the doubtful X_1_X_2_Y in *Lycosa* sp. (Navia et al., 2006). 

Furthermore, studies based on chromosome banding in Lycosidae are still scarce and usually revealed small amounts of heterochromatin at pericentromeric region as observed by Brum-Zorrilla and Cazenave (1974), Chemisquy et al. (2008) and Dolejš et al. (2011). In *Lycosa*, target of this study, only two (*L. erythrognatha* Lucas, 1836 and *L. thorelli* Keyserling, 1877) out of the 15 valid taxa and 19 undefined species (*Lycosa* sp.) with cytogenetic reports (Araujo *et al.*, 2021) have C-band data, also showing small amounts of constitutive heterochromatin (Brum-Zorrilla and Postiglioni, 1980; Chemisquy *et al*., 2008). 

In addition to C-banding, Chemisquy et al. (2008) analyzed the distribution of heterochromatin in Lycosidae using base-specific fluorochromes, and found heterogeneous results among the species, comprising three general patterns, as follows: a) C-band positive and GC-rich pericentromeric heterochromatin as observed in *L. erythrognatha* (Chemisquy *et al*., 2008); b) C-band positive and AT-rich pericentromeric heterochromatin as reported in *Schizocosa malitiosa*, *L. thorelli*, and *Lycosa* sp. by Brum-Zorrilla and Postiglioni (1980); and c) C-band negative and AT-rich terminal heterochromatin also in *Lycosa* sp. (Brum-Zorrilla and Postiglioni, 1980).

On the other hand, silver nitrate staining was performed by Wise (1983) in *Tigrosa georgicola* (Walckenaer, 1837), cited as *Lycosa georgicola*, and by Dolejš et al. (2011) in *Arctosa cinerea* (Fabricius, 1777), *A. lutetiana* (Simon, 1876), *Xerolycosa miniata* (C.L. Koch, 1834), and *X. nemeralis* (Westring, 1861) to identify the nucleolar organizer regions (NORs). In those reports, two NOR-bearing chromosome pairs were invariably observed. 

Subsequently, Forman et al. (2013) identified in *Wadicosa fidelis* (O. Pickard-Cambridge, 1872), by silver nitrate staining and fluorescent *in situ* hybridization (FISH) with 18S rDNA probes, three and seven to ten NOR sites, respectively.

Based on these data, the goal of this study was to extend the chromosomal information in this group of spiders by including refined cytogenetic analyzes. Therefore, different chromosome banding techniques were applied to three *Lycosa* species from Paraná state, Brazil, to investigate karyotypic variability, presence of different SCS, heterochromatin distribution patterns and chromosome mapping of 18S rDNA sites. Based on a comparative approach, we discuss some of the mechanisms that could account for the karyotype differentiation in Lycosinae species.

## Material and Methods

Cytogenetic analyses were performed in three species of *Lycosa*, collected in five locations along the state of Paraná (Table 1). The specimens were deposited in the arachnological collection of the Laboratory of Zoological Collections at the Butantan Institute (IBSP, curator A.D. Brescovit) in São Paulo/SP, Brazil. Chromosomal preparations were obtained according to Araujo et al. (2008), using young and adult spider testicles. The slides were stained with Giemsa 3%, and approximately 30 mitotic and meiotic cells from each individual were analyzed to determine the diploid number. The morphology and chromosome measurements were performed on 10 mitotic metaphases in the Image J program (Schneider et al., 2012) with the use of the plugin LEVAN (Sakamoto and Zacaro, 2009), according to the methodology described by Levan et al. (1964). The slides were submitted to C banding according to Sumner (1972), modified by Lui et al. (2012). The staining with base-specific fluorochromes chromomycin A_3_ (CMA_3_) and 4,6’-diamidino-2’phenylindol (DAPI) was carried out according to Schweizer (1980).

**Table 1 -:** Species analyzed and collection sites. IBSP = Collections Laboratory Zoos, Butantan Institute (curator AD Brescovit), São Paulo / SP, Brazil; PR = Paraná; PEMG=Mata dos Godoy State Park; PNS=Superagui National Park; UEL= State University of Londrina; PNIG=Ilha Grande National Park; PNI = Iguaçu National Park.

Species	Samples (♂)	Voucher (IBSP)	Collection location
*Lycosa erythrognatha* Lucas, 1836	3	216040, 216042, 216043	PEMG - Londrina, PR (23°26’22.92”S 51°14’26.66”W) PNS - Guaraqueçaba, PR (25°21’4.31”S 48°12’3.27”W) UEL - Londrina, PR (23°19’27.07”S 51°12’5.78”W) PNI - Foz do Iguaçu, PR (25°35’17.48”S 54°28’22.09”W)
	15	215907, 216036, 216038, 216044, 216048, 216049, 216078, 216088, 216092, 216093, 216094, 216100, 216106, 216108, 271347	
	4	166431, 166432, 166433, 166437	
	3	166402, 166403, 166405	
*Lycosa* gr. *nordenskjoldi* Tullgren, 1905	3 4	215873, 215874, 271348 242146, 242148, 271349, 271350	PNS - Guaraqueçaba, PR (25°21’4.31”S 48°12’3.27”W) PNIG - Icaraíma/Porto Camargo-PR (43º 22’ 01,00”S 73º 46’ 25,40”W)
*Lycosa sericovittata* Mello-Leitão, 1939	2	166438, 167415	UEL - Londrina, PR (23°19’27.07”S 51°12’5.78”W)

Fluorescent *in situ* hybridization (FISH) was performed according to Schwarzacher and Heslop-Harrison (2000). The 18S rDNA probes were obtained from *Ctenus ornatus* (Keyserling, 1877) by Rincão et al. (2017), labeled with biotin by using the Biotin-Nick Translation Mix (Roche) kit and detected with Avidin-FITC (Invitrogen). Finally, the FISH slides were analyzed using an epifluorescence microscope (Leica DM2000), equipped with digital camera Moticam Pro 282B. The images were captured using the program Motic Images Advanced, version 3.2.

## Results

**Figure 1 -:**
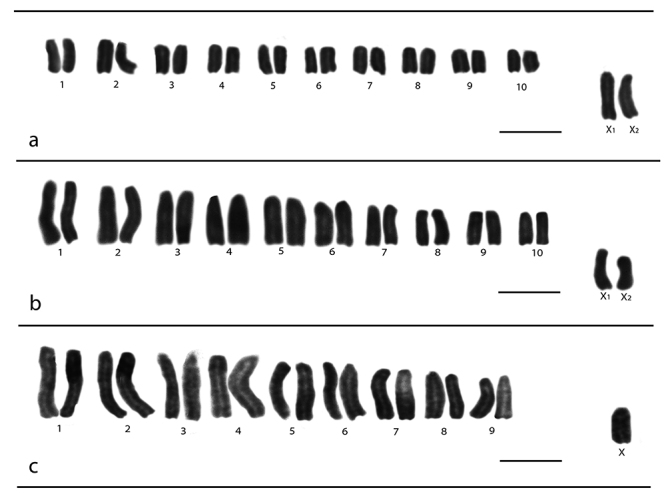
Karyotypes of males of *Lycosa* species stained with Giemsa**. A** *Lycosa erythrognatha* , 2n♂= 22, X_1_X_2_0 of the Mata dos Godoy State Park, State University of Londrina, Iguaçu National Park and Superagui National Park. **B** *Lycosa sericovittata* , 2n♂= 22, X_1_X_2_0 of the State University of Londrina. **C** *Lycosa* gr. *nordenskjoldi*, 2n♂= 19, X0 of the Superagui National Park and Ilha Grande National Park. Bar= 10 μm.

*Lycosa erythrognatha* and *L. sericovittata* presented 2n♂= 22 and SCS X_1_X_2_0 (Figure 1A, B, respectively), while *L.* gr. *nordenskjoldi* presented 2n♂= 19 and SCS X0 (Figure 1C); The karyotypes of the three species are entirely composed of acrocentric chromosomes. However, each species showed different sizes of the sex chromosomes: X_1_ and X_2_ elements are the two largest chromosomes (6.96 - 6.74 µm respectively) in *L. erythrognatha*, where the other chromosomes vary from 5.65 - 3.18 µm; in *L. sericovittata* X_1_ is a medium-sized chromosome of 3.82 µm, significantly distancing itself from the first and largest pair (6.30 µm) and X_2_ is one of the smallest elements (2.69 µm), the other pairs vary 6.05 - 2.94 µm. On the other hand, the X chromosome is the smallest element (3.33 µm) in the karyotype of *L.*gr. *nordenskjoldi*, and the other chromosomes vary from 6.48 - 3.88 µm (Figure 1).

**Figure 2 -:**
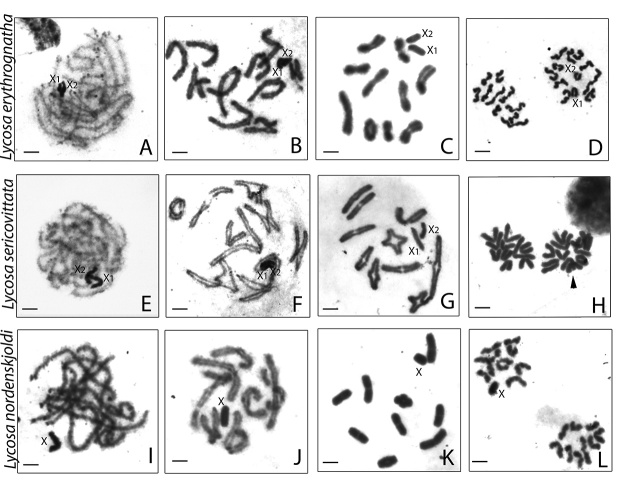
Meiotic cells from *Lycosa* males stained with Giemsa. **A-D** ****Lycosa erythrognatha;* E-H *Lycosa sericovittata;**** I-L *Lycosa* gr*. nordenskjoldi.* Cells in pachytene (**A, E, I**) evidence the positive heteropycnotic sex chromosomes. Diplotene cells showing sexual univalents arranged side by side **(B, F)** or as an isolated univalent **(J);** all cells showing chiasmata, mostly terminal in autosomes. Diakinesis cells confirmed the number of bivalents and sex chromosomes in each species: (**C**) *L. erythrognatha* e (**G**) *L. sericovittata* with 10 autosomal bivalents + X_1_X_2_0 e (**K**) *L.* gr. *nordenskjoldi* with 9 autosomal bivalents + X0. Metaphase II cells show joint migration of sex chromosomes (**D, H**) by observing cells with 12 and 10 chromosomes; and the migration of the single sex chromosome (**L**) showing cells with 10 and 9 chromosomes. The arrowheads in (**H**) indicate the cell with the highest chromosome number. Bar = 10 μm.

In meiotic cells of males from the three species, the sex chromosomes are easily visible in the pachytene nucleus due to their high condensation, being frequently observed as single mass of positive heteropycnosis (Figure 2A, E, I). The diplotene cells in *L. erythrognatha* and *L. sericovittata* (Figure 2B, F, respectively) showed 10 autosomal bivalents and two sexual univalents (10II+X_1_X_2_), whereas *L.* gr. *nordenskjoldi* (Figure 2J) presented 9 autosomal bivalents and one sexual univalent (9II+X), with predominance of terminal chiasmata in the three species. In diakinesis, sexual univalent arranged side by side or very close to each other were observed in both species with X_1_X_2_0 SCS (Figure 2C, G).

Metaphase II cells showed 10 and 12 chromosomes in *L. erythrognatha* (Figure 2D) and *L. sericovittata* (Figure 2H), with the sexual univalents segregation to the same pole, confirming the X_1_X_2_0 SCS. In *L.* gr. *nordenskjoldi* (Figure 2L) 10 and 9 chromosomes were detected in cells during metaphase II, thereby confirming the X0 SCS.

**Figure 3 -:**
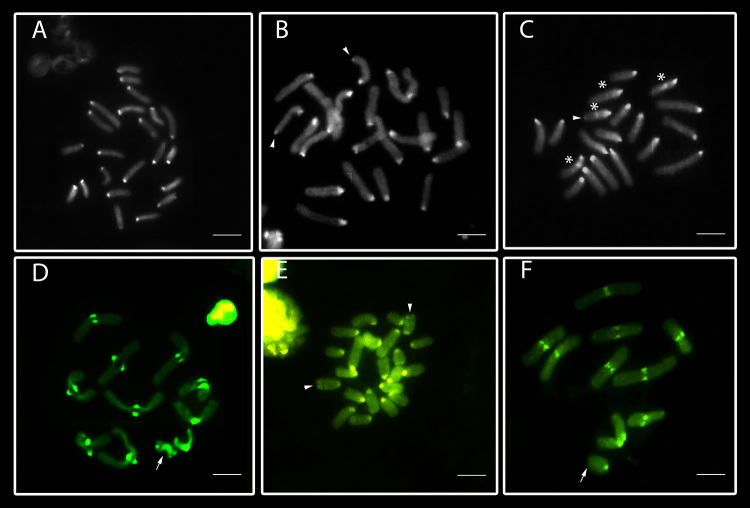
Testicular cells of the three *Lycosa* species submitted to the C banding technique (**A, B, C**) and staining with fluorochrome CMA_3_ **(D, E, F**). The arrows indicate the sex chromosomes. In **A, B, C,** mitotic metaphases of *L. erythrognatha* with 2n♂= 22, X_1_X_2_0 of the Mata dos Godoy State Park, State University of Londrina, Iguaçu National Park and Superagui National Park.*, L. sericovittata* with 2n♂= 22, X_1_X_2_0 of the State University of Londrina, *L.* gr*. nordenskjoldi* with 2n♂= 19, X0 of the Superagui National Park and Ilha Grande National Park *respectively*, showing pericentromeric heterochromatin on all chromosomes. In **(B)** some terminal heterochromatic markings (arrowheads) and in **(C)** interstitial heterochromatin (asterisk). In **(D)** diakinesis cell of *L. erythrognatha* with CMA_3_^+^ pericentromeric regions. Mitotic metaphase of *L. sericovittata* **(E)** with CMA_3_^+^ pericentromeric regions on all chromosomes: arrowheads indicate a pair of chromosomes with terminal markings. In **(F)** diplotene cell of *L.* gr. *nordenskjoldi* showing CMA_3_^+^ pericentromeric markings on all chromosomes. Bar = 10 μm.

The C-banding applied to testicular cells of the three species (Figure 3A, B, C), showed pericentromeric heterochromatin in all chromosomes. Moreover, *L. sericovittata* also showed a chromosome pair with terminal C-bands (Figure 3B) while *L.* gr. *nordenskjoldi* presented some interstitial C-bands regions (Figure 3C). 

The base-specific fluorochrome staining revealed interspecific differences: *Lycosa erythrognatha* and *L. gr. nordenskjoldi* (Figure 3D, F, respectively) presented CMA_3_^+^ pericentromeric signals in all chromosomes, coinciding with heterochromatic regions in the former. In addition to the pericentromeric CMA_3_^+^ regions, *L. sericovittata* also presented GC-rich sites at terminal regions of two chromosomes (Figure 3E). No DAPI^+^ signals (AT-rich sites) were detected in the analyzed species (data not shown).

**Figure 4 -:**
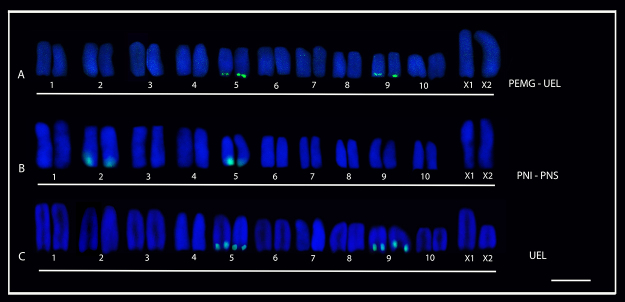
Karyotypes of males of two *Lycosa* species submitted to fluorescent *in situ* hybridization (FISH) with 18S rDNA probe. In (**A**) and **(B)** *L. erythrognatha:* in (A) from Mata dos Godoy State Park (PEMG) and State University of Londrina (UEL), showing the sites of 18S rDNA in pairs 5 and 9; in (**B**) from Iguaçu National Park (PNI) and Superagui National Park (PNS) showing ribosomal sites in pairs 2 and 5; in (**C**) *L. sericovittata* from UEL, with sites of 18S rDNA in pairs 5 and 9. Bar = 10 μm.

The FISH experiments revealed four 18S rDNA sites in *L. erythrognatha*, with interpopulation variation of 18S-bearing pairs. Therefore, these ribosomal cistrons were located in pairs 5 and 9 of three individuals from Mata dos Godoy State Park (PEMG) and the four specimens from the State University of Londrina (UEL) (Figure 4A), while 10 individuals from Superagui National Park (PNS) and three from Iguaçu National Park (PNI) presented positive signals in pairs 2 and 5 (Figure 4B). In *L. sericovittata,* FISH also identified four 18S rDNA sites, at the terminal region of pairs 5 and 9 of the two individuals analyzed (Figure 4C). 

**Figure 5 -:**
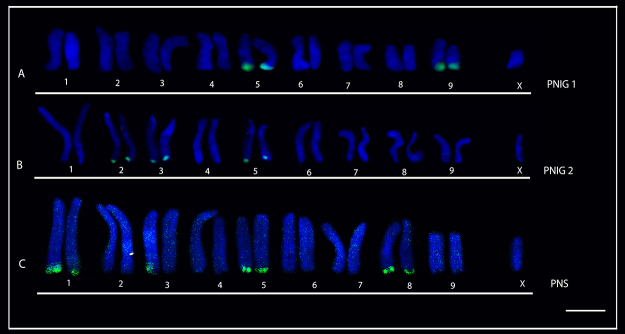
Karyotypes of two populations of *L.* gr. *nordenskjoldi* submitted to fluorescent in situ hybridization (FISH) with 18S rDNA. (**A**) individual from the Ilha Grande National Park (PNIG) population with ribosomal sites in pairs 5 e 9; (**B**) individual from the PNIG population with ribosomal sites in pairs 2, 3 e 5; (**C**) Superagui National Park (PNS) population with sites of 18S rDNA in pairs 1, 3, 5 and 8; pair 3 shows marking in only one of the chromosomes. Bar = 10 μm.

In addition, inter and intrapopulational variation in the number of 18S rDNA was observed in *L.* gr. *nordenskjoldi,* ranging from four, six and seven 18S signals. Similarly, this species also presented variation in the pairs bearing ribosomal sites, as follows: two individuals from Ilha Grande National Park (PNIG) showed four 18S rDNA sites at terminal region of pairs 5 and 9 (Figure 5A) while two individuals from the same locality presented six sites in pairs 2, 3 and 5 (Figure 5B). On the other hand, the three samples from PNS were characterized by seven 18S rDNA sites at terminal region of pairs 1, 5 and 8, and on a single chromosome from pair 3 (Figure 5C).

## Discussion

### Karyotype analysis

The diploid numbers, presence of SCS and karyotypes composed of acrocentric chromosomes observed in *L. erythrognatha*, *L. sericovittata*, and *L.* gr. *nordenskjoldi* were consistent with previous reports in these species (Diaz and Saez, 1966; Chemisquy et al., 2008; Araujo et al., 2015); however, only *L.* gr. *nordenskjoldi* was previously analyzed in another population of the state of Paraná (Araujo *et al*., 2015). The occurrence of acrocentric or telocentric chromosomes is regarded as a common trait shared among species of Entelegynae (Dolejš et al., 2011; Araujo *et al*., 2015).

Four congeneric species (*L. chaperi* Simon, 1885, *L. thorelli* Keyserling, 1877, *L. carmichaeli* Gravely, 1924 and *L. pampeana* Holmberg, 1876) also exhibited the pattern reported in *L. erythrognatha* and *L. sericovittata*, i.e., 2n♂=22, X_1_X_2_0 (Mittal, 1966; Brum-Zorrilla and Postiglioni, 1980; Srivastava and Shukla, 1986; Chemisquy et al., 2008). On the other hand, only *L.* gr. *nordenskjoldi* and *Hogna sternalis* (Bertkau, 1880) presents 2n ♂ = 19, X0 (Araujo *et al*., 2021; present study), indicating that this is a rare diploid number within spiders of the family Lycosidae. Even though SCS X_1_X_2_0 has been commonly reported in Lycosidae, being considered an ancestral condition in spiders (Araujo *et al*., 2016), the presence of 2n = 22 is found in less than 20% of species in this family. Whereas most of them are characterized by 2n = 28 (Araujo *et al*., 2021). Within the genus *Lycosa* genus, the available karyotypic analyzes demonstrated high frequencies of both 2n values, followed by a less frequent occurrence of diploid numbers, ranging from 18 to 27 chromosomes (Araujo *et al*., 2021). 

It should be pointed out that species of *Lycosa* show considerable levels of chromosomal variation in spite of the low number of species analyzed so far. This feature and the fact that this genus is recognized as a polyphyletic group composed of many species, jeopardizes reliable estimates about the ancestral diploid number in *Lycosa* and their karyoevolutionary relationships.

### Patterns of heterochromatin distribution

The three species analyzed shared the common pattern of heterochromatin distribution described by Chemisquy et al. (2008), including C-bands and GC-rich segments at pericentromeric regions. However, variations were found in these species, such as the presence of GC-rich terminal sites in a specimen of *L. sericovittata*. Moreover, *L.* gr. *nordenskjoldi* was characterized by heterogeneity of heterochromatin distribution due to the occurrence of pericentromeric, interstitial and terminal C-bands while GC-rich sequences were restricted to the pericentromeric region. Therefore, two additional patterns of C-banding were identified in this study: (1) the presence of terminal GC-rich heterochromatin segments; and (2) interstitial heterochromatin with no signs of GC or AT richness (CMA_3_^-^/DAPI^-^).

The distribution of heterochromatic blocks at pericentromeric regions, had been considered as distinctive feature within Lycosidae, as supported by the data reported by Chemisquy et al. (2008) and the present results. Nevertheless, our data demonstrated novel patterns of heterochromatin distribution in this group of spiders in which *L.* gr. *nordenskjoldi* stands out by the high dispersal of heterochromatin segments. A comparative analysis between these results and the putative ancestor pattern of heterochromatin distribution in *Lycosa* suggests that paracentric inversions or dispersal of repetitive sequences could be related to the C-banding pattern described in *L.* gr. *nordenskjoldi*.

The variability in the data obtained by C-banding and fluorochrome staining, particularly in *Lycosa* gr. *nordenskjoldi*, in addition to the diploid number, confirms that traditional chromosomal markers allow differentiating congeneric species, at least in comparison with data described in literature so far. 

### Interpopulation chromosomal variability of 18S rDNA sites

Despite the presence of two 18S rDNA-bearing pairs in *L. erythrognatha*, this species showed interpopulation variation in the position of these sites in different chromosomes in the karyotypes. The Superagui National Park (PNS), located on the northern coast of the state of Paraná, is a region of islands and mangroves with a more tropical climate, similar to that of the Iguaçu National Park (PNI) in the western boundary of Paraná, which includes one of the largest conserved areas of Atlantic Forest in Brazil. The populations of *L. erythrognatha* from both regions (PNS and PNI) exhibited a different karyotypic pattern in relation to those from the northern region (PEMG and UEL), a region of dry climate and characterized by semideciduous seasonal forest vegetation. In spite of the geographic distance between these locations (PNS and PNI), which is about 560 km, we infer that adaptive processes in these populations should be comparable to each other because they share similar habitats. Analogously, the environmental differences among the four populations should impose differential selective pressure, thereby determining distinct evolutionary pathways.

This interpopulation variation in 18S rDNA-bearing pairs may be related to gene dispersal via transpositions or translocations, as suggested by Cabral de Melo *et al*. (2011) in a study with beetles (Scarabaeinae). These authors point out that in the absence of significant karyotypic changes (e.g., increase or decrease in diploid numbers), the ribosomal sites can disperse and vary as a result of successive amplification processes of these cistrons, particularly when located at distal portion of chromosomes inasmuch as these regions are considered highly dynamic, thus favoring the dispersal of rDNA copies throughout the genome. 

The distribution of ribosomal sites in *L.* gr. *nordenskjoldi* showed both inter and intrapopulation variation. The variability between the two populations (PNS and PNIG) of this species also can be related to their habitat. Despite being similar to each other, the evolutionary pressure can act in different ways on populations from distinct species, eventually resulting in independent accumulation of chromosomal rearrangements in locally adapted individuals, as previously reported in *Wadicosa fidelis* (Forman et al., 2013) and in harvestmen species (*Opiliones*, Phalangiidae) (Šťáhlavský et al., 2018).

On the other hand, the presence of 18S rDNA sites in pairs 5 and 9 was shared by the three species, with pair 5 observed in all populations, despite the variability in location and number of ribosomal cistrons. Apparently, this would be a conserved trait in these species what remains to be confirmed by further studies, since this is the first report based on FISH experiments in *Lycosa*. 

### Chromosomal diversification within Lycosinae

Recent phylogenetic inferences (Piacentini and Ramiréz, 2019), revealed that most species of Lycosidae from south America represent undescribed genera, what should explain the karyotypic diversity observed in literature and in the present work. Furthermore, the South American species usually present lower diploid numbers than Eurasian representatives (Araujo *et al*., 2021).

In addition, the phylogenetic reconstruction presented by Piacentini and Ramiréz (2019), Lycosinae encompasses species from North and South American, as well as the genus *Hogna* and Eurasian species *of Lycosa.* The latter, along with the outgroup Pardosinae, has a predominance of species with 2n = 28, X_1_X_2_0 (Araujo *et al.*, 2021), which can be suggested as the ancestral diploid number of the Lycosinae subfamily.

Only six out of the total of cytogenetically analyzed species in Lycosinae (Araujo *et al*., 2021) are characterized by changes in the diploid number involving sex chromosomes, as follows: *Hogna sternalis* (Bertkau, 1880) - 2n♂ = 19, X0; *Lycosa barnesi* Gravely, 1924-2n♂ = 27, X0 (Eurasian region); *L.* gr. *nordenskjoldi* Tullgren, 1905-2n♂ = 19, X0; *Lycosa* sp. - 2n♂ = 21, X_1_X_2_Y; *Lycosa* (*thorelli* group) -2n♂ = 23, X_1_X_2_X_3_0; and *Schizocosa* (*malitiosa* group) - 2n ♂ = 23, X0. 

Analyzing the data presented in Araujo *et al.* (2021), these unusual diploid numbers were determined by alterations in the SCS, related to increases or decreases in the number of chromosomes with the consequent evolution of new SCS. As mentioned earlier**,** X_1_X_2_0 SCS is regarded as an ancestral condition for several groups of spiders, including Lycosoidea (Dolejš et al., 2011; Araujo *et al*., 2015). Thus, other SCS systems should be considered as derived features. Some studies, including those by Král *et al*. (2006) and Araujo *et al*. (2012, 2014), have previously demonstrated that X_1_X_2_X_3_0 and X_1_X_2_0 SCS coexist within a single genus or, even, in the same species (Araujo *et al.*, 2014; Rincão et al., 2020). 

The origin of the above mentioned SCSs in Entelegynae was hypothesized by several authors as follows: 1) by fusions or fissions in the sex chromosomes during the conversion of X_1_X_2_0 to X_1_X_2_X_3_0 system and vice versa, and during the conversion of X_1_X_2_0 to a single X0 system (Pätau, 1948; Postiglioni and Brum-Zorrilla, 1981; Parida and Sharma, 1986); 2) by the formation of a supernumerary element during the conversion of X_1_X_2_0 to X_1_X_2_X_3_0 system (Bole-Gowda, 1952); and 3) by fusions between sex and autosomal chromosomes, especially during the conversion of X_1_X_2_0 to X_1_X_2_Y system (Král *et al*., 2006). Despite this great variability in SCS, Entelegynae spiders share two notable characteristics, which are the predominance of acrocentric chromosomes and the occurrence of X_1_X_2_0 SCS (Král *et al*., 2006; Araujo et al., 2014), which is observed in Lycosinae.

One of the most cited chromosomal rearrangements is the fusion between autosomes and sex chromosomes, as proposed by Hackman (1948), resulting in metacentric elements, usually followed by pericentric inversions or partial deletion (Datta and Chatterjee, 1989, 1992). Another event often hypothesized within this context would be the in tandem fusion, resulting in the origin of acrocentric chromosomes (Pekár and Král, 2001). When changes in diploid number take place without modifications in the X_1_X_2_0 SCS, rearrangements such as single translocation or in tandem fusion among autosomal chromosomes are inferred, thus maintaining the acrocentric/telocentric chromosomal set. Such event might have caused the differentiation of karyotypes with 2n♂ = 26, 24, 22 and 18. These diploid numbers are reported, for example, in *Gladicosa pulchra* (Keyserling, 1877); *Lycosa madani* Pocock, 1901; *Schizocosa malitiosa* (Tullgren, 1905); and *Lycosa tarantula* (Linnaeus, 1758), respectively.

On the other hand, when changes in both diploid numbers and SCS occur, the even diploid number is modified into an odd chromosome number. In this case, an X_1_X_2_0 SCS originates the novel and less frequent systems: X0, X_1_X_2_X_3_0 and X_1_X_2_Y, present in some species of *Lycosa* (South America) associated with distinctive morphology of sex chromosomes. Accordingly, the X_1_X_2_X_3_0 system could have arisen from the insertion of a supernumerary chromosome in the former X_1_X_2_0 SCS (Bole-Gowda, 1952) or from chromosomal non-disjunction (Postiglioni and Brum-Zorrilla, 1981; Datta and Chatterjee, 1988). Additionally, the X_1_X_2_Y SCS could emerge after a translocation between sex and autosomal chromosomes (Silva et al., 2002; Rowell, 2004; Král *et al*., 2006, 2007).

Therefore, the presence of lower diploid numbers and unusual SCS is likely to derive from karyotypes with similar 2n values instead of a series of fusions in former karyotypes with 2n ♂ = 28. For example, the occurrence of 2n♂ = 23, X_1_X_2_X_3_0 should rather evolve from 2n ♂ = 22, X_1_X_2_0 by the formation of a supernumerary element than through multiple fusion/fission events.

Otherwise, the karyotypes with 2n♂ = 27, X0; 2n♂ = 23, X0; and 2n♂ = 19, X0 would have emerged through fusions between sex chromosomes, followed by a putative pericentric inversion. These derived SCSs are present in many other spider families along with other systems, but they have been rarely reported (Král *et al*., 2006; Araujo et al., 2012, 2014). 

In conclusion, this study demonstrated a wide variation in chromosomal features among and within the three species of *Lycosa*, as evidenced by the differences in both number and location of 18S rDNA sites and heterochromatic blocks, especially in the species complex *Lycosa* gr. *nordenskjoldi*. The data also showed that genomes have undergone chromosomal breaks and translocation/chromosome fusions, which account for the differentiation of diploid numbers and sex chromosomes system in species of Lycosinae.
